# Exploring the Remarkably High Photocatalytic Efficiency of Ultra-Thin Porous Graphitic Carbon Nitride Nanosheets

**DOI:** 10.3390/nano14010103

**Published:** 2024-01-01

**Authors:** Zahra Kalantari Bolaghi, Cristina Rodriguez-Seco, Aycan Yurtsever, Dongling Ma

**Affiliations:** Centre Énergie Materiaux et Telécommunications, Institut National de la Recherche Scientifique (INRS), Varennes, QC J3X 1P7, Canada; zahra.kalantari@inrs.ca (Z.K.B.); aycan.yurtsever@inrs.ca (A.Y.)

**Keywords:** photodegradation, hydrogen evolution reaction, porous carbon nitride, exfoliation

## Abstract

Graphitic carbon nitride (g-C_3_N_4_) is a metal-free photocatalyst used for visible-driven hydrogen production, CO_2_ reduction, and organic pollutant degradation. In addition to the most attractive feature of visible photoactivity, its other benefits include thermal and photochemical stability, cost-effectiveness, and simple and easy-scale-up synthesis. However, its performance is still limited due to its low absorption at longer wavelengths in the visible range, and high charge recombination. In addition, the exfoliated nanosheets easily aggregate, causing the reduction in specific surface area, and thus its photoactivity. Herein, we propose the use of ultra-thin porous g-C_3_N_4_ nanosheets to overcome these limitations and improve its photocatalytic performance. Through the optimization of a novel multi-step synthetic protocol, based on an initial thermal treatment, the use of nitric acid (HNO_3_), and an ultrasonication step, we were able to obtain very thin and well-tuned material that yielded exceptional photodegradation performance of methyl orange (MO) under visible light irradiation, without the need for any co-catalyst. About 96% of MO was degraded in as short as 30 min, achieving a normalized apparent reaction rate constant (*k*) of 1.1 × 10^−2^ min^−1^mg^−1^. This represents the highest *k* value ever reported using C_3_N_4_-based photocatalysts for MO degradation, based on our thorough literature search. Ultrasonication in acid not only prevents agglomeration of g-C_3_N_4_ nanosheets but also tunes pore size distribution and plays a key role in this achievement. We also studied their performance in a photocatalytic hydrogen evolution reaction (HER), achieving a production of 1842 µmol h^−1^ g^−1^. Through a profound analysis of all the samples’ structure, morphology, and optical properties, we provide physical insight into the improved performance of our optimized porous g-C_3_N_4_ sample for both photocatalytic reactions. This research may serve as a guide for improving the photocatalytic activity of porous two-dimensional (2D) semiconductors under visible light irradiation.

## 1. Introduction

The exponential rise in urban populations, rapid economic progress, and long-term industrial and agricultural activities have caused a severe energy crisis and produced harmful pollutants that led to climate change. This is having a tremendous negative effect on both human health and the environment [1,2,3]. Specifically, the discharge of waste materials, such as organic dyes, surfactants, chloro-organic compounds, or pesticides, from certain industries became a significant threat to public health, aquatic life, and ecosystems. The main causes include their toxic nature, persistence in the environment, and their ability to alter natural habitat balance and harm numerous living organisms [4,5,6]. Furthermore, the increased global utilization of fossil fuels in the past decades is directly related to human economic and social development. However, it is provoking a severe energy crisis, additional pollution, and an acceleration of global warming. To tackle above-mentioned problems, we need to seek more environmentally friendly technologies that lower the energy requirements and production costs [7,8,9].

To date, several traditional methods have been utilized, including adsorption [10], filtration [11], and electrochemical processes [12]. However, they fall short in completely getting rid of organic pollutants and require high energy consumption and cost [13]. Solar-involved photocatalysis stands out as a highly efficient technology that converts abundant solar energy into chemical energy in the presence of a semiconductor catalyst. It can be used for various applications, including hydrogen production [14,15], CO_2_ reduction [16,17], N_2_ fixation [18,19], and water purification [20,21], among others. Compared to previously mentioned methods, photocatalysis has lower energy and operating costs, is more sustainable, and requires minimal use of chemicals [22]. Additionally, the materials used in this process are more stable, reducing the need for frequent replacement [23,24]. In a photocatalytic reaction, electron–hole pairs are generated in a semiconductor upon illumination. These electrons and holes migrate to the photocatalyst surface to take part in redox reactions. Numerous semiconductors, including TiO_2_ [25], ZnS [26], ZnO [27], CdS [28], and Cu_2_O [29], have been widely used in the past few decades for pollutant degradation and hydrogen production. However, their photocatalytic efficiency is low, limiting their practical application. This is due to their wide bandgap, which decreased photo-response range and visible light-harvesting capabilities, and fast photoinduced charge carrier recombination [30,31,32]. Thus, it is imperative to seek out new strategies and alternative materials that allow us to efficiently utilize solar energy and improve photocatalytic activity.

Carbon nitride (C_3_N_4_) has gained significant interest as a photocatalyst in the field of pollutant degradation and hydrogen production due to its visible photoactivity and other outstanding properties [33,34,35,36]. C_3_N_4_ is a metal-free semiconductor that possesses visible absorption, has a layered structure, shows great thermal and chemical stability, and features low-cost and easy synthesis [37,38,39]. Its band gap (2.7 eV) and valence and conduction band edges (1.3 eV and −1.4 eV versus standard hydrogen electrode (NHE), respectively) make it suitable for water splitting, degradation of organic pollutants, and CO_2_ reduction [40,41]. However, its practical applications are hindered because of its still limited absorption in the visible range, a high recombination rate of photogenerated charge carriers, and a low specific surface area caused by its bulk nature [42,43,44]. To address its shortcomings, numerous efforts have been made, including metal and non-metal doping [45], defect engineering [46], surface modification with other carbon-based nanomaterials [47], morphology modulation [48], and coupling with other lower-bandgap semiconductors [49]. Nevertheless, improving the photocatalytic performance of C_3_N_4_-based materials to a suitable level, without involving any expensive metals, has remained a significant challenge [50,51].

The two-dimensional (2D) nanosheet structure enhances the photocatalytic activity of g-C_3_N_4_, with respect to its bulk counterpart, by significantly improving its light absorption and increasing its surface area [52,53]. Further reducing the thickness of the nanosheets to make ultra-thin materials with few layers, typically below 10 nm in thickness, can further reduce charge carrier diffusion length, boost its light absorption, lower the recombination rate, and provide more exposed active sites for closer interface contact between the photocatalyst and pollutants [54,55]. Additionally, ultra-thin materials can be further modified by attaching functional groups onto their surface to fine-tune their electronic, optical, and chemical properties [39]. A useful technique for separating stacked layers of g-C_3_N_4_ into thinner ones is through exfoliation, such as ball milling, and chemical, ultrasonication, and thermal treatments [56,57]. The process of chemical exfoliation usually involves the use of oxidizing acids such as nitric acid (HNO_3_) and sulfuric acid (H_2_SO_4_) [58,59]. During this process, H^+^ intercalates between layers of C_3_N_4_ using nitrogen as acceptor sites, which causes crystal swelling, increases the interlamellar distance, and decreases sheet attraction [60,61]. As a result, g-C_3_N_4_ nanosheets become separated. For example, Xu et al. [62] synthesized ultra-thin g-C_3_N_4_ nanosheets using chemical exfoliation with H_2_SO_4_. They obtained single-layered nanosheets with a large surface area and superior transport of photogenerated charge carriers, enhancing their photocatalytic activity by 2.6 times when compared to bulk C_3_N_4_. It has also been demonstrated that the use of HNO_3_ during the chemical exfoliation process introduces N vacancies and increases the possibility that O atoms replace the mentioned vacancies simultaneously. This modifies the density of the photoinduced electrons and holes, the band gap, and the visible light absorption in the photocatalyst [63,64]. Zhang et al. [65] revealed that the introduction of cyano groups and N vacancies into porous C_3_N_4_ resulted in 26 times higher hydrogen production compared to bulk C_3_N_4_. This was caused by defects that improve charge separation efficiency and promote its light-harvesting capacity. In another study, Niu et al. [66] demonstrated that N vacancies introduced to bulk C_3_N_4_ can lead to an increase in light absorption between 450 nm and 600 nm and a narrowing of the band gap from 2.74 eV to 2.66 eV. Ultrasonication in liquid media creates microbubbles and powerful jets, highly increasing pressure and temperature locally. These ultrasonic waves break down the weak van der Waals forces of g-C_3_N_4_ layers, leading to the formation of nanosheets [67,68]. The presence of dangling hydrogens among multilayer C_3_N_4_ makes polar solvents, such as water, ethanol, and ammonia, more effective for ion intercalation [69]. Zhang et al. [70] used ultrasonication to exfoliate bulk C_3_N_4_ in water, yielding thin sheets of 70–160 nm in size and 2.5 nm in height (equivalent to seven layers of C-N). These sheets showed enhanced photo-absorption that translated into an increased photocurrent and a three times higher photocatalytic activity when compared to bulk C_3_N_4_, for Rhodamine B (RhB) photodegradation in the presence of H_2_O_2_.

In addition, the incorporation of pores into g-C_3_N_4_ nanosheets is expected to increase specific surface area, surface active sites, and charge separation [71,72]. Altering the pore configuration and size distribution of porous g-C_3_N_4_ nanosheets is a successful approach for enhancing its optoelectronic properties and photocatalytic efficacy [73]. For example, Zhang et al. [74] synthesized mesoporous g-C_3_N_4_ using HCl and ethanol, and obtained higher specific area and pore volume that yielded a greater activity for the degradation of RhB (degradation percentage of 99.9% in 210 min) compared to that of g-C_3_N_4_ (degradation percentage of 61.4% in 240 min).

In this study, we synthesized ultra-thin porous g-C_3_N_4_ nanosheets, by means of thermal exfoliation and ultrasonication in an acid medium. We achieved a substantial time and energy reduction during the exfoliation step using acid when compared to prior publications that required long hours of stirring in acid media, or a high temperature [75,76,77,78]. Moreover, the addition of HNO_3_ and ultrasonication greatly modified the chemical and physical properties of porous g-C_3_N_4_-based samples. We present here a rigorous study of how the synthetic steps affect the photocatalytic performance of our materials when used for pollutant degradation and hydrogen production by thoroughly analyzing their light absorption, structure, morphology, and pore size distribution. With that, we were able to achieve a normalized apparent reaction rate constant (*k*) of 1.1 × 10^−2^ min^−1^mg^−1^, as well as providing physical insights into materials engineering towards achieving high visible-driven photocatalysis performance of a very promising material based on g-C_3_N_4_.

## 2. Materials and Methods

**Scheme 1:**
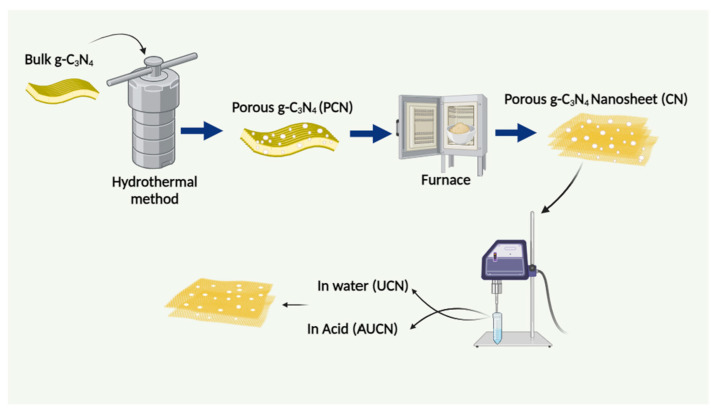
Schematic diagram of the synthetic procedure of ultra-thin porous g-C_3_N_4_ nanosheets.

Melamine (C_3_H_6_N_6_, 99%), nitric acid (HNO_3_), 1,4-benzoquinone (BQ), tert-butyl alcohol (t-BuOH), and disodium ethylenediaminetetraacetate (Na_2_EDTA) and TEOA (10 vol %) were bought from Sigma-Aldrich Corporation (Oakville, ON, CA) and utilized without any additional processing. 

g-C_3_N_4_ samples were synthesized by changing several conditions (i.e., temperature, sonication time, sonication solution) until the optimal sample (thermally treated at 500 °C, 5 min ultrasonication in acid) was obtained (Scheme 1). Bulk C_3_N_4_ (BCN) was first prepared by heating melamine in a muffle furnace by gradually increasing temperature with a heating rate of 2.5 °C min^−1^ under air. Upon reaching 550 °C, heating continued for 4 h and then it gradually cooled down to room temperature. The synthesis of g-C_3_N_4_ nanosheets involved a multi-step method, including a hydrothermal treatment that yielded the formation of a porous structure, and different exfoliation steps to finally obtain ultra-thin nanosheets. First, 0.9 g of BCN was added to 60 mL of deionized water and sonicated for 20 min using a sonication bath. The dispersion was then poured into a 125 mL Teflon-lined stainless-steel autoclave and heated in an oven at 180 °C for 12 h. The mixture was centrifuged and air-dried at 80 °C overnight. The produced powder was named as porous g-C_3_N_4_ (PCN). To form porous nanosheets, the as-prepared PCN sample (0.5 g) was put in a crucible and heated up in a furnace at a heating rate of 5 °C min^−1^ in air and then thermally treated at different temperatures (500 °C, 550 °C, and 600 °C) for 2 h. The corresponding samples were named 500-CN, 550-CN, and 600-CN, respectively. To synthesize ultra-thin porous g-C_3_N_4_ nanosheets, chemical and ultrasonic exfoliation were further used. The as-prepared samples (0.1 g of 500-CN, 550-CN, and 600-CN) were added to 20 mL of diluted HNO_3_ (~8 M). Then, they were sonicated using tip sonication for 5 min. Finally, the samples were washed with water and ethanol to remove potential alkaline residues. Some samples were synthesized via ultrasonic exfoliation only in water for 5 min (500-UCN, 550-UCN, and 600-UCN), and others, as mentioned earlier, via ultrasonic exfoliation for 5 min in the presence of HNO_3_ (500-AUCN, 550-AUCN, and 600-AUCN). A summary of all the prepared samples is shown in Table 1.

An XPS analysis was conducted using a VG Escalab 220i-XL instrument equipped with a twin-anode (Mg/Al) source. To adjust for charging, the binding energies (BEs) were calibrated against the N1s peak at 299 eV. A CHI 660E electrochemical workstation was used for photoelectrochemical (PEC) measurements in a standard three-electrode cell. The counter and reference electrodes were a Pt wire and an Ag/AgCl electrode (3 M KCl), respectively. The working electrode was made on FTO glass and protected by Scotch tape. The electrolyte solution employed was a pre-purged 0.2 M aqueous Na_2_SO_4_ solution (pH = 6.8), with nitrogen purging carried out for 30 min beforehand. A Newport solar simulator with intensity of 100 mW/cm^2^ was utilized as the light source. Nyquist plots were acquired with a frequency range of 100 mHz to 100 kHz at a bias of 0.4 V. In addition, through the diffuse reflectance spectroscopy (DRS) technique (Perkin Elmer, Lambda 750), the optical characteristics of porous g-C_3_N_4_ nanosheets were investigated. The Varian Cary 5000 scan spectrometer was used to measure MO absorbance in an aqueous solution. A PANalytical X’Pert MRD instrument was employed to evaluate the crystal structures of all samples. A Cu Kα radiation source (λ = 0.15406 nm) was used at 45 kV and 40 mA. The Brunauer–Emmett–Teller (BET) instrument model TMAXCN (TMAX-BSD-PM2) was employed to investigate pore size distribution and surface area of porous materials. The photocatalysts’ morphology was investigated using a transmission electron microscope (TEM, JEOL 2100F) operating at 200 kV. 

The photocatalytic activity of g-C_3_N_4_ was evaluated using MO dye, a common contaminant in water, as a model reagent. A typical experiment involved adding 10 mg of the sample into a 100 mL quartz reactor containing a solution of 25 mL of MO (10 mg/L). The mixture was stirred in the absence of light for 30 min to establish an adsorption–desorption equilibrium before being exposed to illumination. A 300 W Xenon lamp positioned 20 cm away from the solution was utilized in this experiment. A long-pass optical filter of 400 nm was used to allow only visible light to pass through. In total, 0.9 mL of MO was sampled throughout various time intervals and centrifuged to separate from the powder. A UV-vis absorption spectrometer was used to determine the absorbance of MO in the supernatant. As our reaction follows the first-order reaction model, the apparent reaction rate constant (*k*_app_) was estimated using the equation ln(C_0_/C) = *k*_app_ t, where t is reaction time, and C_0_ and C are the MO concentrations at time 0 and t, respectively. The obtained *k*_app_ value was then normalized with the entire mass of the photocatalysts, referred to as the normalized apparent reaction rate constant (*k*) for performance comparison. 

To carry out a photocatalytic hydrogen evolution reaction (HER), a top-irradiation Pyrex reaction vessel covered with a quartz glass connected to a closed gas system was used. The process involved dispersing 50 mg of the catalyst in 45 mL of water. In total, 5 mL of TEOA (10 vol %) was added as the sacrificial agent. Platinum (1.0 wt %) was loaded onto the surface of the catalyst through an in situ photo deposition method using H_2_PtCl_6_·6H_2_O as a precursor. To deposit platinum on samples after sonication of the mixture for 5 min, irradiation with a 300 W Xenon lamp, using a full spectrum, was used while stirring for 1 h. Then, the reactor was sealed and vacuumed before being irradiated by a 300 W Xenon lamp for HER. The incident light’s wavelength was controlled via an appropriate long-pass cut-off filter (λ > 400 nm). The suspension was continuously stirred and maintained at constant room temperature by circulated cooling water. The evolved gases were analyzed at 60 min time intervals by sampling 1 mL of gas from the reactor and injecting it into a gas chromatograph (GC, 7890B, Agilent Technologies, Mississauga, ON, CA) equipped with a thermal conductivity detector.

## 3. Results and Discussion

In this work, we performed systematic sample optimization by treating bulk g-C_3_N_4_ using different conditions and following certain procedures. First, PCN was synthesized from BCN through the hydrothermal treatment, forming a porous structure. Then, for the optimization of the porous samples, thermal exfoliation at various temperatures (500 °C, 550 °C, and 600 °C) was employed as a way to obtain thinner nanosheets. The optimal temperature (500 °C) was selected based on the photocatalytic performance, which was affected by the pore size, functional surface groups, and thin layered structure. Ultimately, we used tip sonication in water and HNO_3_ media to obtain ultra-thin-based samples, to introduce defects and functional groups, and increase light absorption. Typical samples involved in the optimization process are listed in Table 1. Numerous characterization tests were carried out to understand how the various conditions affected the structural, morphological, optical, electrical, and photocatalytic properties of final samples. 

**Figure 1:**
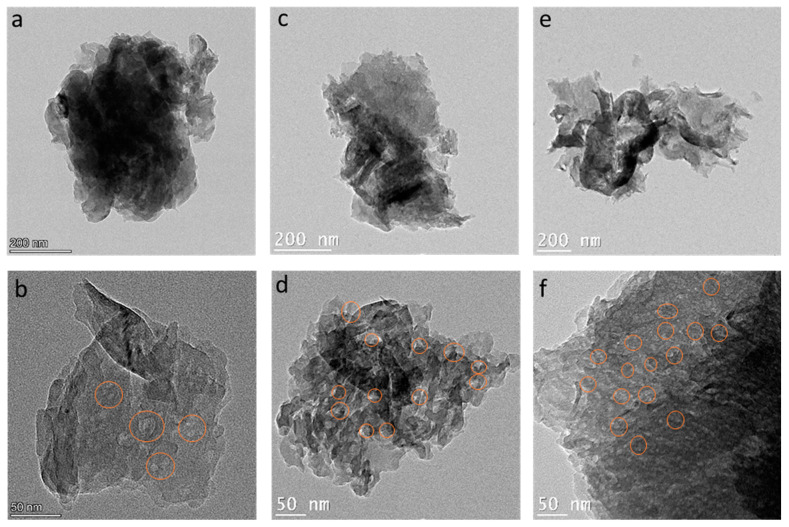
TEM images of (**a**,**b**) 500-CN, (**c**,**d**) 500-UCN, (**e**,**f**) 500-AUCN. Orange circles highlight formed pores in these samples.

TEM was performed to analyze the structure and morphology of g-C_3_N_4_. Figure 1 shows typical low and relatively higher magnification TEM images of resulting mesoporous nanosheets exfoliated at 500 °C after ultrasonication in water and acid. It can be observed that sample 500-CN has more layers of g-C_3_N_4_ stacked together with larger pore sizes (Figure 1a,b). Similar stacked structures were also observed for 550-CN and 600-CN (Figure S1a,c). After performing sonication (500-UCN), the number of layers decreases, and nanosheets break at the larger pores, creating smaller nanosheets with smaller pores (Figure 1c,d). Upon sonication in HNO_3_ (500-AUCN), g-C_3_N_4_ displays much smaller pores (~5 nm) and thinner layers due to etching by strong oxidizing acid and ultrasonication together (Figure 1e,f). The nanosheets’ breakdown and exfoliation become more noticeable with longer sonication time in an acid medium (Figure S2a,b). Upon comparing Figure 1a,c,e, it becomes evident that ultrasonication in water and acid leads to a notable reduction in the thickness (i.e., the number of layers) of the nanosheets, thereby signifying the successful exfoliation of C_3_N_4_. TEM images for samples exfoliated at 550 °C (550-CN) and 600 °C (600-CN) are shown in Figure S1. The sample treated at 550 °C (Figure S1b) presents a larger pore size than samples exfoliated at 500 °C, possibly decreasing its specific surface area. From Figure S1d, it can be clearly observed that the nanosheets crumbled into small and non-uniform fragments when the thermal treatment temperature increased to 600 °C (sample 600-CN). Also, from its photocatalytic activity, we observed a much lower performance compared to 500-CN and 550-CN. Its poor activity (Table S1) was attributed to its greater pore size and the loss of its nanosheet structure. The photocatalytic performance of 550-CN was also lower than that of 500-CN (Table S1). Therefore, we decided to focus our study on samples treated at 500 °C.

**Figure 2:**
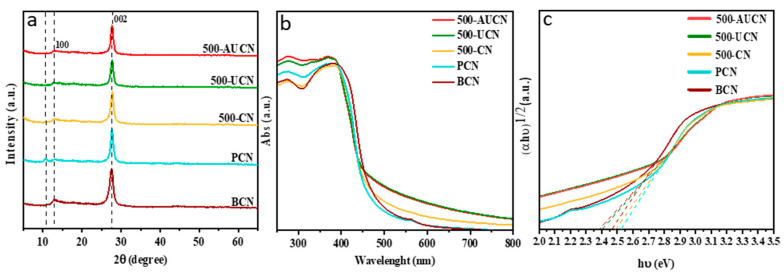
(**a**) XRD patterns, (**b**) DRS spectra, and (**c**) Tauc’s plot for optical bandgap (E_g_) analysis of synthesized g-C_3_N_4_ samples.

XRD was employed to investigate the structural characteristics of synthetized samples. As illustrated in Figure 2a, the interlayer stacking of conjugated aromatic C–N heterocycles is indicated by the strong (002) diffraction peak at 27.4°, present in all the samples. The interplanar tri–s–triazine units are represented by the (100) diffraction peak located at 13.0° [79,80]. After the hydrothermal step performed on BCN, we observed the appearance of an extra peak at ~10.7°. This peak has been attributed to the slight change in alignment of the tri-s-triazine unit arising from the oxidation/hydrolysis of g-C_3_N_4_ during the hydrothermal treatment [81,82]. It disappears after the thermal exfoliation, possibly due to the delamination of nanosheets [83]. An enlarged view of the (002) peak is shown in Figure S3. It shows that BCN has a higher intensity of the (002) peak in comparison to other samples. This supports the successful exfoliation of the samples, as evidenced by TEM images (Figure 1). Based on the XRD analysis, we can conclude that the thermal exfoliation and the ultrasonication steps (either in water or acid media) did not significantly affect the interlayer nor interplanar structure of the photocatalysts, as this was also demonstrated elsewhere [84,85].

The photocatalytic activity of a material is governed by three main processes: surface redox reactions, the separation and transfer of charges, and light absorption [86,87,88]. To measure the optical absorption, we conducted UV-vis DRS tests and used the results to estimate the band gap energy of our samples. Figure 2b shows that samples 500-UCN and 500-AUCN (after ultrasonication in water and acid media) present an increased light absorption in both visible and UV regions. This phenomenon could be due to the improved separation and reduced agglomeration of g-C_3_N_4_ layers resulting from ultrasonication and acid exfoliation, or because of the defect states’ absorption process [89,90,91]. 550-AUCN shows a further increase in its absorption onset (486 nm), being the sample with the highest and broadest absorption in the visible and UV regions (see Figure S4). The greater utilization of photons could be caused by its improved layered structure after being treated at a higher temperature during the thermal step at 550 °C [92]. In addition, at higher temperatures, the increase in the condensation of N and C atoms leads to the expansion of electron delocalization in the aromatic nanosheets, which is the cause of the enlarged absorption [93]. Figure 2c shows the Tauc’s plot (solid lines) derived from the UV-vis DRS measurements and linear extrapolation (dashed lines). The obtained values for the band gaps were 2.39 eV, 2.40 eV, 2.49 eV, 2.52 eV, and 2.46 eV for 500-AUCN, 500-UCN, 500-CN, PCN, and BCN, respectively. It can be observed that both ultrasonication in acid and water resulted in a decrease in the bandgap with respect to 500-CN and PCN, similar to other published work [94]. Previously, it was found that the introduction of structural defects and oxygen-containing functional groups could narrow the band gap of C_3_N_4_ [46,95,96]. In particular, N vacancies normally produce mid-gap states in the band gap close to the conduction band, thus narrowing it [97]. We believe it is also the case in our samples, i.e., introduced N vacancies and oxygen-containing functional groups were responsible for the narrowing of the band gap of 500-AUCN and 500-UCN. On the other hand, 500-CN (2.49 eV) and PCN (2.52 eV) samples showed an increase in band gap energy compared to BCN (2.46 eV), which is due to the quantum confinement effect and could strengthen their redox ability [70,98]. 

For the photocatalytic reaction to happen, in the first step of the process, pollutants must adsorb onto the surface of the photocatalyst. This is where photogenerated charge carriers initiate redox reactions that will contribute to pollutants’ degradation [99,100]. Thus, to enhance the photocatalytic activity, it is important to analyze the surface area and the textural characteristics of the photocatalysts through gas adsorption tests and using the BET theory. The surface area is 61.3 m^2^/g, 62.8 m^2^/g, 53.3 m^2^/g, 53.6 m^2^/g, and 8.82 m^2^/g for 500-AUCN, 500-UCN, 500-CN, PCN, and BCN, respectively. Samples that have undergone ultrasonication in water and acid media (500-UCN and 500-AUCN) present similar surface area (62.8 and 61.3 m^2^/g, respectively), but it is much larger than that of BCN (~7 times). The trend goes as follows: 500-UCN ≈ 500-AUCN > 500-CN ≈ PCN >>> BCN. This suggests that the hydrothermal treatment was able to significantly increase the specific surface area by creating pores, and ultrasonication, whether in water or acid, could further enlarge the specific surface area through mechanical exfoliation. The greater surface area of 500-UCN and 500-AUCN is in line with the enhanced light absorption observed in Figure 2b [98,101]. In addition, 500-AUCN had a smaller average pore size in comparison to the other samples (Figure S5), agreeing with our TEM analysis shown in Figure 1. A general feature was that ultrasonication caused a considerable boost in the number of micro-pores (<2 nm) in 500-UCN and 500-AUCN, while 500-CN had larger pores with fewer micro-pores, in comparison to other porous samples. Based on these characterizations, we proposed that the thermal treatment resulted in thermal etching and an enlarged pore size. In addition, the subsequent sonication step caused the breakdown through larger pores into smaller and thinner nanosheets with more micro-pores and small mesopores (see Figure S5 and Figure 1). But importantly, the sonication time needs to be optimized because excessive ultrasonication, especially in acid media (500-AUCN—40 min), caused a collapse in the structure of g-C_3_N_4_ and its pores (Figure S2), and a large performance drop. 

**Figure 3:**
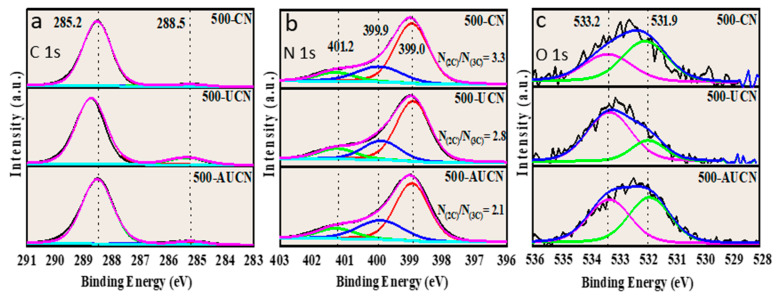
(**a**) C 1s, (**b**) N 1s, and (**c**) O 1s XPS spectra of g-C_3_N_4_ samples exfoliated at 500 °C.

XPS was carried out to further explore the changes in the chemical state, elemental composition, and surface functional groups of the as-synthetized samples. Two C1s peaks at 285.2 eV and 288.5 eV were observed (Figure 3a), attributed to the sp^2^ C=C bonds of the graphitic C and the adventitious hydrocarbons, and sp^2^ C in N–C=N bonds, respectively [102,103]. The deconvolution of N1s peaks (Figure 3b) revealed three distinct components positioned at 401.2 eV, 399.9 eV, and 399.0 eV, corresponding to the N-H_x_ group in the heptazine framework, bridging N in -N-(C)_3_ (N_3C_), and sp^2^ hybridized N in -C-N=C or C-N=C=(N_2C_), respectively [104,105]. N_(2C)_/N_(3C)_ ratios for 500-AUCN, 500-UCN, and 500-CN were 2.1, 2.8, and 3.3. The reduced ratio of N_(2C)_/N_(3C)_ in 500-AUCN suggests the creation of and increase in two-coordinated N vacancies with respect to more stable three-coordinated N atoms [106], perhaps due to the powerful oxidizing properties of nitric acid, further aided through ultrasonication. Figure 3c shows high-resolution spectra for O1s. The two peaks located at 531.9 eV and 533.2 eV are attributed to -C=O/C-O and -OH groups [107,108]. 500-UCN clearly exhibits a higher concentration of -OH groups and 500-CN has a higher concentration of -C=O/C-O, while 500-AUCN shows a similar concentration of -C=O/C-O and -OH groups. Moreover, from the XPS survey spectra, the concentration of oxygen-containing groups in 500-AUCN was the highest among the three samples, being ~1.3 and 2 times that in 500-UCN and 500-CN, respectively, again supporting the fact that the considerable oxidization reaction occurred during the ultrasonication-in-acid process [109]. It suggests that this process led to not only smaller pore sizes but also obvious variations in chemical species.

**Figure 4:**
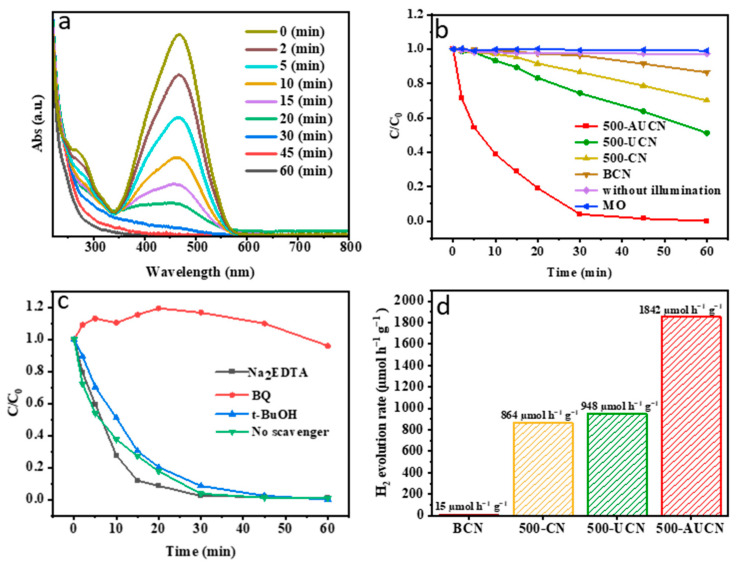
(**a**) UV-vis absorption spectra of MO during the photodegradation process using 500-AUCN, (**b**) photocatalytic activity test using different g-C_3_N_4_ samples, (**c**) photocatalytic degradation of MO under visible light, using 500-AUCN in the presence of three different scavengers, and (**d**) hydrogen evolution rate of samples 500-AUCN, 500-UCN, 500-CN, and BCN under visible light irradiation.

To assess the photocatalytic activity of the samples, MO degradation was performed under visible light irradiation (λ > 400 nm) at room temperature. Figure 4a displays the UV-vis absorption spectra of MO during the photodegradation reaction in the presence of 500-AUCN. The MO absorption peak at ~464 nm dramatically decreased to almost zero, indicating its nearly complete degradation after 30 min. Figure 4b shows the MO photodegradation percentage as a function of the reaction time for different samples. 500-AUCN had the greatest activity (red line in Figure 4b), removing 96% of MO in 30 min, and reaching 99% after 1 h, whereas only 50%, 30%, and 13% of MO were removed in 1 h when using 500-UCN, 500-CN, and BCN, respectively. Two control tests were carried out to better assess the photocatalytic performance, (i) MO under visible light irradiation without any photocatalyst and (ii) MO in the presence of the most effective material (500-AUCN), but under dark conditions. No degradation of MO occurred in either of the control tests, suggesting MO removal is not caused by the photocatalyst or light alone, and its degradation is due to the photocatalytic reaction in the presence of our C_3_N_4_-based materials. The *k* values (summarized in Table 2) show the trend of 500-AUCN (11.2 × 10^−3^ mg^−1^ min^−1^) > 500-UCN (2.3 × 10^−3^ mg^−1^ min^−1^) > 500-CN (1.4 × 10^−3^ mg^−1^ min^−1^) > BCN (0.2 × 10^−3^ mg^−1^ min^−1^). Impressively, the *k* value for 500-AUCN is 58 times higher than that of BCN. The MO photodegradation using the photocatalyst samples exfoliated at 550 °C and 600 °C followed the same trend (Figures S6–S8 and Table S1). It is clear that ultrasonication in acid media is beneficial to photocatalysis regardless of the exfoliation temperature (500 °C, 550 °C, and 600 °C). The experiment was conducted four times for the best sample (500-AUCN) over a total duration of 240 min (Figure S9). The photocatalyst exhibited high efficiency in removing MO and demonstrated good stability for photocatalytic applications. Moreover, we would like to remark that the sample 500-AUCN presented the highest photocatalytic performance for MO degradation among all the C_3_N_4_-based materials reported so far (Table S2). We link this to the oxidizing nature of HNO_3_, which, during the sonication process, generated N vacancies and oxygen-containing functional groups in g-C_3_N_4_ that can help photocatalysis.

To comprehend the mechanism involved in the degradation of MO, we tested several trapping agents for our benchmark sample, 500-AUCN (Figure 4c). Specifically, t-BuOH, BQ, and Na_2_EDTA were used to capture h^+^, •O_2_^−^, and •OH, respectively. It was found that the addition of Na_2_EDTA and t-BuOH had almost no impact on the degradation of MO, which means that •OH and h^+^ did not play a major role in the photocatalytic process. On the other hand, when BQ was added, the photocatalytic activity was notably reduced, suggesting that •O_2_^−^ acted as the primary species responsible for the degradation of MO.

We also tested hydrogen production under visible light for all the as-synthetized samples. As shown in Figure 4d, 500-AUCN once again showed the best performance, with a hydrogen production rate of 1842 µmol h^−1^ g^−1^, 120 times higher than that of BCN (15 µmol h^−1^ g^−1^). Samples 500-UCN and 500-CN also displayed higher photocatalytic activity when compared to BCN, with HER of 948 µmol h^−1^ g^−1^ and 864 µmol h^−1^ g^−1^, respectively. 

**Figure 5:**
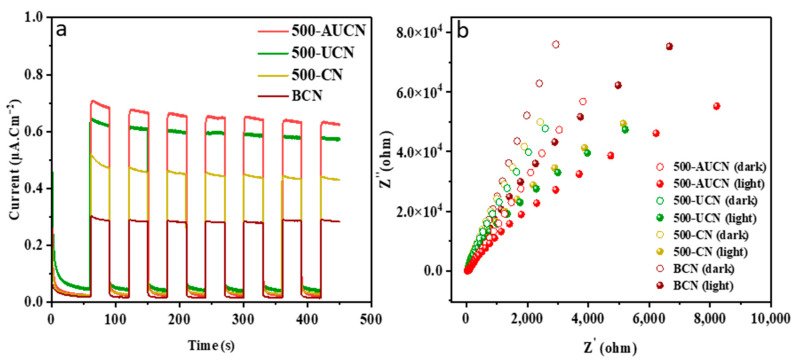
(**a**) Transient photocurrent response of g-C_3_N_4_ samples under a solar simulator and (**b**) EIS Nyquist plots in the dark and under simulated solar light irradiation of g-C_3_N_4_ samples.

We also subjected the 500 °C exfoliated samples and BCN to PEC tests and consistently we observed that 500-AUCN and 500-UCN (the ones that underwent sonication in acid and water, respectively) have a greater photocurrent generation compared to 500-CN and BCN (Figure 5a). This suggests that improved samples have a better charge carrier generation and/or transfer to the surface, which could result in a more beneficial photocatalytic activity. Figure 5b displays the results of Electrochemical Impedance Spectroscopy (EIS) measurements on multiple samples. The EIS Nyquist plots of the 500-AUCN and 500-UCN samples show a smaller arc radius when compared to 500-CN and BCN, both in the dark and under irradiation. This indicates that the charge transfer resistance in the former two samples is lower, possibly due to the presence of more functional groups.

The differences in performance of all samples synthesized in this work are related to their optical, chemical, and surface properties. In this section, we aim to provide a comprehensive analysis of the key properties exhibited by the most efficient sample, while highlighting the modifications that were introduced during the synthetic process. Our goal is also to assist readers in gaining a better understanding of the enhanced photocatalytic behavior, allowing researchers to rationally design and synthesize C_3_N_4_-based materials. The analysis is performed from the following aspects:

I. Optical properties:

Light absorption has a noticeable influence on the semiconductor’s performance. 500-AUCN and 500-UCN exhibited a broader light absorption profile within the visible spectrum due to their smaller bandgaps caused by the introduction of defects through ultrasonication in water and acid. Broader light absorption tends to favor the generation of a higher number of electron–hole pairs when subjected to light irradiation, thereby leading to improved photocatalytic properties [110]. This is in agreement with Zhang et al.’s work, where they also found an increased absorption of C_3_N_4_ in the visible region when ultrasonication was performed [70].

II. Chemical properties:

The excellent photocatalytic performance of 500-AUCN for MO degradation is highly related to the nature of its surface functional groups (-C=O/C-O and -OH) generated through the ultrasonication step in nitric acid. They potentially act as active sites increasing the photocatalytic activity [111]. 500-AUCN showed a higher number of oxygen-containing functional groups based on our XPS studies. According to previous studies [112,113], adding -C=O/C-O functional groups can improve the photocatalytic activity of g-C_3_N_4_ by enhancing the hydrophilicity of its surface, the number of active sites, and the effectiveness of charge separation. -OH groups could boost the electron transfer in C_3_N_4_ and prevent aggregation of layers by forming interlayer hydrogen bonds, thus improving photocatalytic activity [114]. This could at least serve as one reason for the best performance of 500-AUCN for hydrogen production as well. A hydrogen evolution reaction mostly relies on efficient light absorption and charge separation, followed by effective redox reactions to release hydrogen and oxygen from water [115,116]. Ultrasonication in acid helps these factors by creating defects that narrow the bandgap in the structure of g-C_3_N_4_ [117].

Moreover, the ultrasonication step in acid media, performed in the synthesis of 500-AUCN, resulted in a reduction in the number of N_2C_ atoms relative to N_3C_ atoms when compared to 500-UCN and 500-CN, leading to the creation of N vacancies that enhance its photocatalytic performance [64,118]. It has been reported that a higher concentration of nitrogen vacancies improves the separation of photogenerated charge carriers and light absorption [119,120]. Furthermore, N_3C_ has a larger number of unpaired electrons in the nitrogen atoms, resulting in a higher density of active sites available for photocatalytic processes [121]. Wang et al. [104] demonstrated that surface nitrogen vacancies reduce the recombination of photoinduced charge carriers by creating localized electronic trap states within the band gap of the material. This led to an increase in the number of charges that migrated to the photocatalyst surface and participated in following redox reactions to degrade pollutants [117].

III. Surface properties:

It is well known that ultrasonication helps to separate stacked layers to create 2D materials [122,123,124]. When ultrasonication was performed on 500-CN (53.3 m^2^/g), we observed a moderate increase in specific surface area for 500-AUCN and 500-UCN (61.4 m^2^/g and 62.9 m^2^/g, respectively). This potentially provided more exposed active sites that are available for the MO adsorption and redox reaction involved in its photodegradation [125]. 

Furthermore, compared to other samples, 500-AUCN has the smallest pore size. These small and well-distributed pores serve a dual purpose. Firstly, they reduce van der Waals interaction by creating many boundaries. Secondly, they create cross-planar diffusion channels and numerous new active edges. These features accelerate the diffusion and mass transfer of the photogenerated charge carriers to a great extent [126]. The results of a recent study have shown that the size and distribution of pores in porous g-C_3_N_4_ nanosheets play a significant role in the interaction between the semiconductor and organic pollutants during the photocatalytic process [127]. Specifically, microporous and small mesoporous structures, which were also observed in 500-AUCN, are beneficial for the mass transfer and molecular diffusion of reactants to the active sites of the C_3_N_4_ semiconductor [128]. Due to the reduction in pore size and number of stacked nanosheet layers, the number of active sites for photocatalytic reactions increases and bulk diffusion length to the active sites decreases, thereby lowering the probability of recombination among photoexcited charge carriers [129,130].

## 4. Conclusions

We developed an optimized procedure to synthesize porous ultra-thin g-C_3_N_4_ nanosheets that involved the combination of a thermal step at 500 °C and ultrasonication in acid. This method was the most effective in exfoliating C_3_N_4_, yielding ultra-thin nanosheets with high photocatalytic performance. It reduced the time and energy required for exfoliation while producing ultra-thin nanosheets with small, well-distributed pores. We achieved an impressive *k* value of 11.2 × 10^−3^ mg^−1^ min^−1^ for the photodegradation of MO when using 500-AUCN. This is the highest value known to date utilizing C_3_N_4_-based materials for MO degradation. This was due to the combination effect of its porous thin layered structure, increased light absorption, nitrogen vacancies, and oxygen-containing functional groups created through ultrasonication in acid. Hydrogen production was also performed for the as-synthetized samples. The best production rate was also achieved for 500-AUCN (1842 µmol h^−1^ g^−1^). This indicates that this sample is suitable for both applications. This study presents an efficient method for exfoliating C_3_N_4_-based materials, with high photocatalytic performance that paves the way to tackle the organic matter pollution and provides an alternative to non-renewable energy sources.

## Figures and Tables

**Table 1:** Typical samples synthesized during the optimization process.

Sample	Hydrothermal Method	Thermal Exfoliation	Ultrasonication in Acid	Ultrasonication in Water
500-CN	180 °C	500 °C	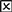	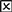
550-CN	180 °C	550 °C	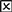	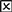
600-CN	180 °C	600 °C	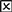	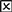
500-UCN	180 °C	500 °C	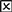	☑
550-UCN	180 °C	550 °C	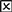	☑
600-UCN	180 °C	600 °C	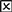	☑
500-AUCN	180 °C	500 °C	☑	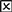
550-AUCN	180 °C	550 °C	☑	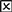
600-AUCN	180 °C	600 °C	☑	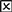

**Table 2:** *k* values for the MO photodegradation reaction in the presence of photocatalytic g-C_3_N_4_ samples exfoliated at 500 °C.

Sample	*k* (10^−3^ mg^−1^ min^−1^)
500-AUCN	11.2 ± 0.35
500-UCN	2.3 ± 0.48
500-CN	1.4 ± 0.30

## Data Availability

Data are contained within the article and Supplementary Materials.

## Supplementary Materials

The following supporting information can be downloaded at: https://www.mdpi.com/article/10.3390/nano14010103/s1, Figure S1: TEM images of thermally exfoliated g-C_3_N_4_ samples: (a and b) 550-CN and (c and d) 600-CN; Figure S2: g-C_3_N_4_ sample ultrasonicated in acid media for 40 min; Figure S3: Enlarged view of (002) peak of XRD for g-C_3_N_4_ samples; Figure S4: DRS spectra of g-C_3_N_4_ samples prepared under different conditions; Figure S5: Pore size distribution curves of different g-C_3_N_4_ samples; Figure S6: Visible light degradation spectra of MO using samples (a) 500-CN, (b) 500-UCN, (c) 550-CN, (d) 550-UCN, (e) 550-AUCN, (f) 600-CN, (g) 600-UCN, and (h) 600-AUCN; Figure S7: Photocatalytic degradation of MO under visible light using photocatalyst samples exfoliated at 550 °C; Figure S8: Photocatalytic degradation of MO under visible light, using photocatalyst samples exfoliated at 600 °C; Figure S9: Photocatalytic stability of 500-AUCN in four successive cycling reactions under visible light irradiation; Table S1: Normalized reaction rate (*k*) values for the MO photodegradation reaction in the presence of photocatalytic samples exfoliated at 550 °C and 600 °C; Table S2: Performance of C_3_N_4_-based samples for degradation of MO under visible light irradiation in recently published literature. References [131,132,133,134,135,136,137,138,139,140,141] are cited in the Supplementary Materials.
