# Supplementary material for: Exploring the Remarkably High Photocatalytic Efficiency of Ultra-Thin Porous Graphitic Carbon Nitride Nanosheets
Source: Nanomaterials (Basel). 2024 Jan 1;14(1):103. doi: 10.3390/nano14010103 (PMC10781176; doi:10.3390/nano14010103)
Supplement: Supplementary file 1 [file nanomaterials-14-00103-s001.zip › nanomaterials-2776574-supplementary.pdf]

Supplementary Materials

Exploring the Remarkably High Photocatalytic Efficiency of Ultra-Thin Porous Graphitic Carbon Nitride Nanosheets

Zahra Kalantari Bolaghi, Cristina Rodriguez-Seco *, Aycan Yurtsever and Dongling Ma *

Centre Énergie Materiaux et Telécommunications, Institut National de la Recherche Scientifique (INRS), Varennes, QC J3X 1P7, Canada; zahra.kalantari@inrs.ca (Z.K.B.); aycan.yurtsever@inrs.ca (A.Y.)

* Correspondence: cristina.rodriguez.seco@inrs.ca (C.R.-S.); dongling.ma@inrs.ca (D.M.)

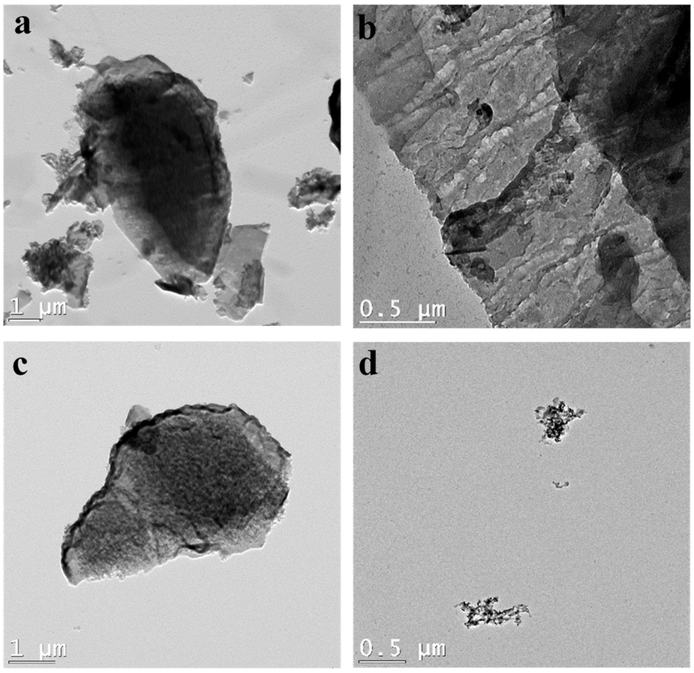

Figure S1. TEM images of thermally exfoliated g-C_3N_4 samples: (**a** and **b**) 550-CN and (**c** and **d**) 600-CN.

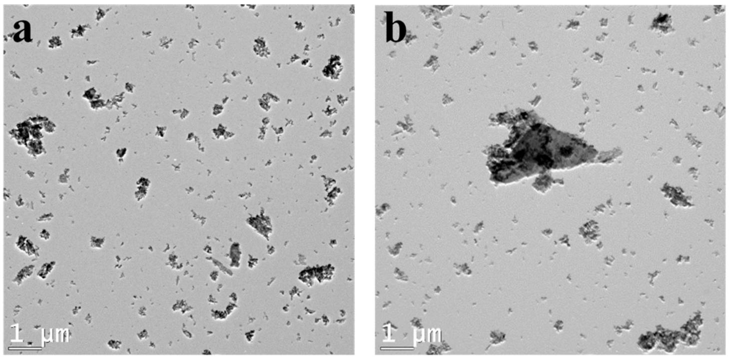

Figure S2. g-C_3N_4 sample ultrasonicated in acid media for 40 min (500-AUCN-40 min).

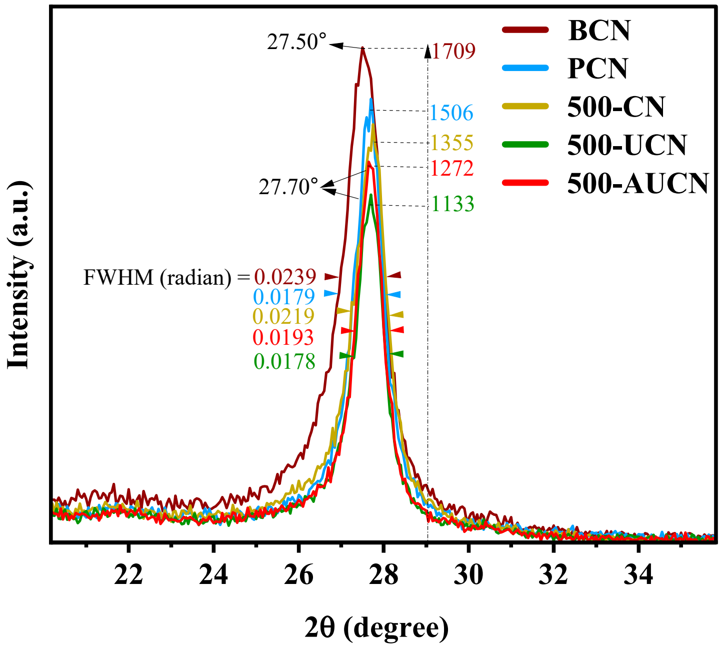

Figure S3. Enlarged view of (002) peak of XRD for g-C_3N_4 samples.

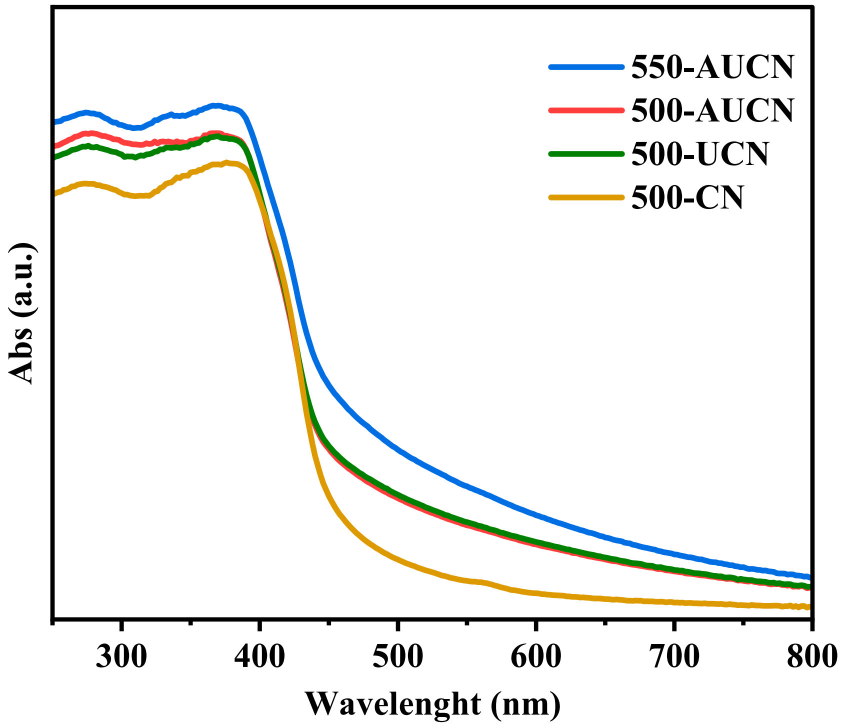

Figure S4. DRS spectra of g-C_3N_4 samples prepared under different conditions.

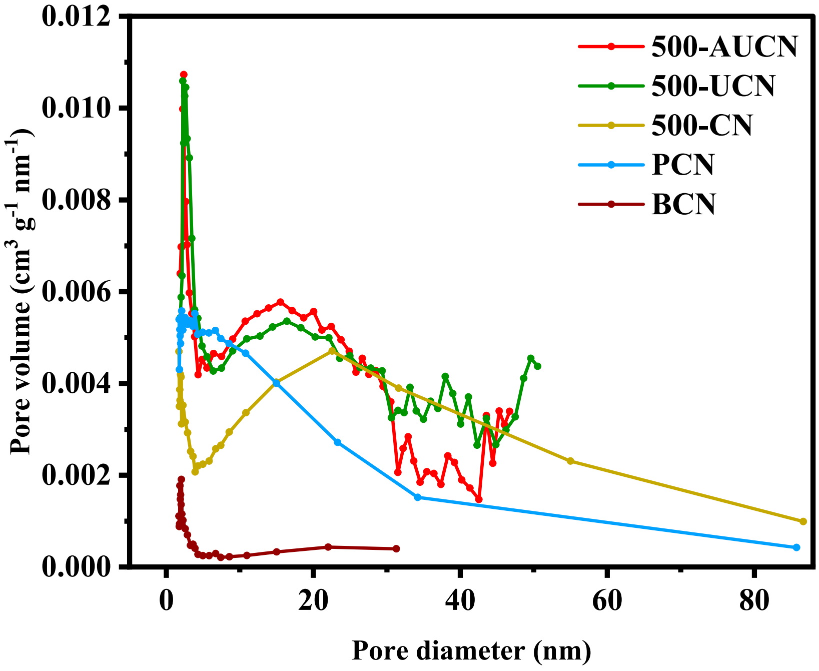

Figure S5. Pore size distribution curves of different g-C_3N_4 samples.

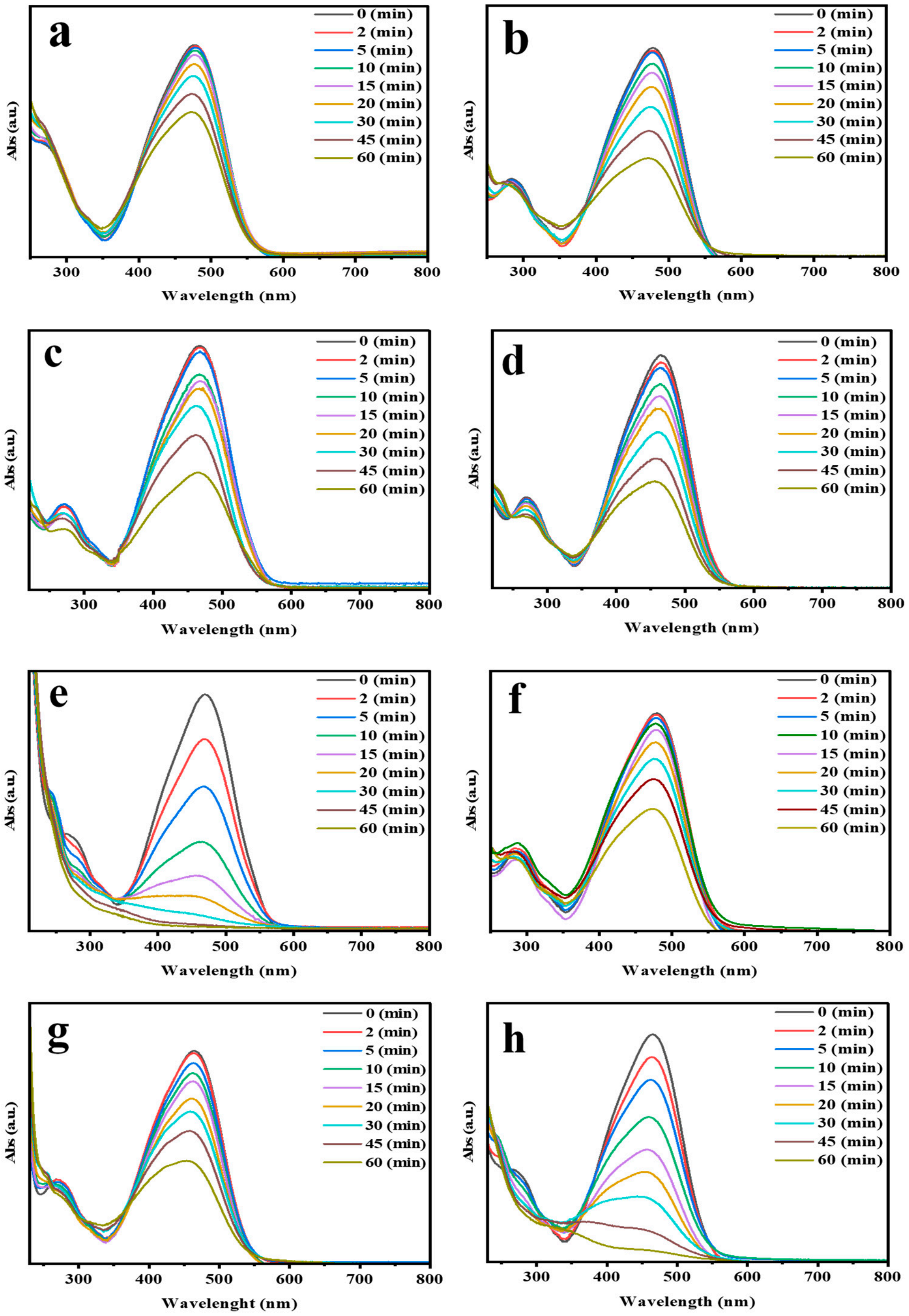

Figure S6. Visible light degradation spectra of MO using samples (a) 500-CN, (b) 500-UCN (c) 550-CN, (d) 550-UCN, (e) 550-AUCN, (f) 600-CN, (g) 600-UCN, and (h) 600-AUCN.

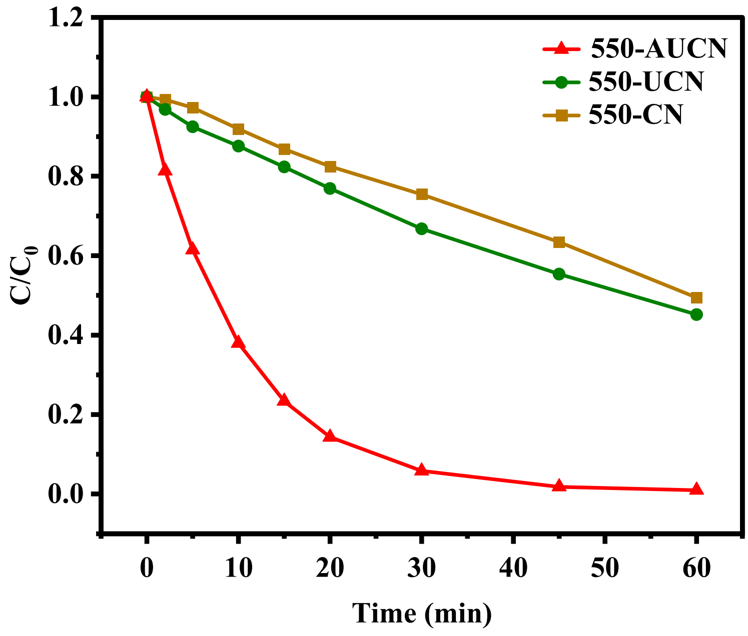

Figure S7. Photocatalytic degradation of MO under visible light using photocatalyst samples exfoliated at 550 °C.

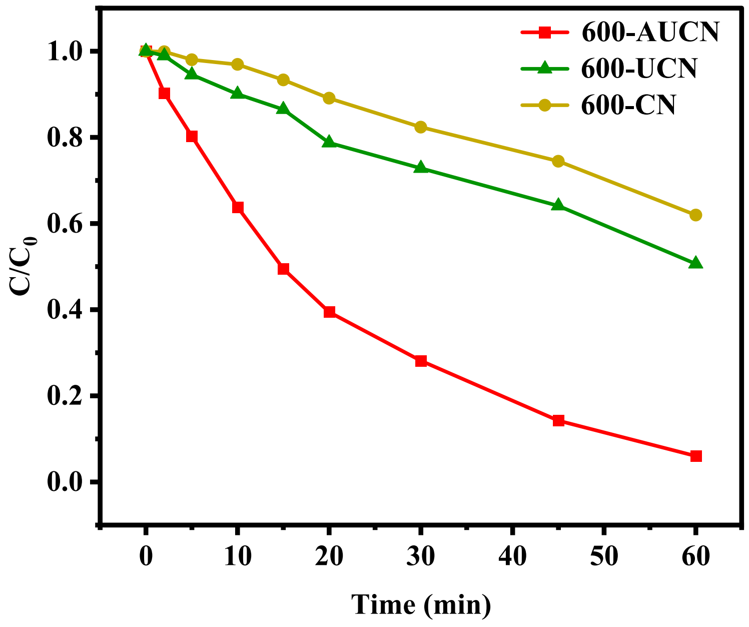

Figure S8. Photocatalytic degradation of MO under visible light, using photocatalyst samples exfoliated at 600 °C.

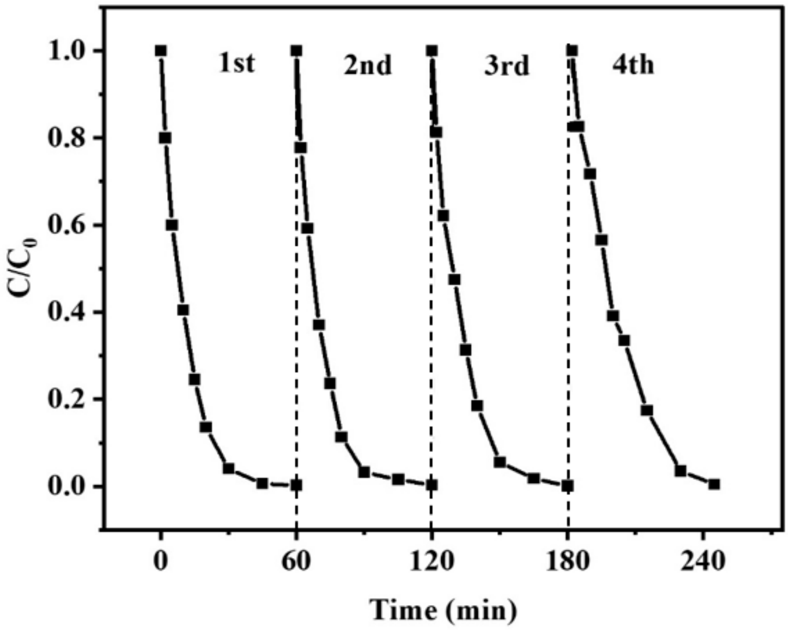

Figure S9. Photocatalytic stability of 500-AUCN in four successive cycling reactions under visible light irradiation.

Table S1. Normalized reaction rate (*k*) values for the MO photodegradation reaction in the presence of photocatalytic samples exfoliated at 550 °C and 600 °C.

Sample	k (10^{-3} min^{-1} mg^{-1})
**550-AUCN**	8.0
**600-AUCN**	4.5
**550-UCN**	1.3
**600-UCN**	1.0
**550-CN**	1.1
**600-CN**	0.7

Table S2. Performance of C_3N_4-based samples for degradation of MO under visible light irradiation in recently published literatures.

References	Photocatalyst	Mass (mg)	Volume (mL)	MO concentration (mg L^{-1})	Light source	k value (10^{-3} min^{-1} mg^{-1})
[1]	Porous Nanosheet	40	80	5	> 420 nm 300 W Xenon lamp	1.95
[2]	Porous Nanosheet	25	50	5	> 420 nm 500 W Xenon lamp	0.31
[3]	PCZ QDs/g-C_3N_4	10	25	10	> 420 nm 300 W Xenon lamp	2.10
[4]	V_2O_5/P-g-C_3N_4	50	50	10	> 420 nm 500 W Xenon lamp	0.43
[5]	Cu/ZnO-g-C_3N_4	50	100	10	Visible light, Mercury lamp	2.80
[6]	Ag/g-C_3N_4	25	100	10	> 420 nm 300 W Xenon lamp	1.00

[7]	Na-doped g-C_3N_4	20	50	10	> 420 nm 500 W Xenon lamp	1.30
[8]	g-C_3N_4/Ag/P_3HT	60	30	10	> 420 nm 100 W LED lamp	0.18
[9]	CNS-TiO_2/g-C_3N_4[a]	20	50	20	> 420 nm 300 W Xenon lamp	3.45
[10]	NG@g-C_3N_4[b]	60	100	10	Visible light	0.45
[11]	MoS_2/Fe_3O_4/g-C_3N_4[c]	40	20	10	Visible light, 60 W LED lamp	6.57
**This work**	Ultrathin Porous Nanosheet	10	25	10	Visible light 300 W Xenon lamp	11.70

[a] carbon–nitrogen–sulfur co-doped TiO_2/g-C_3N_4, [b] N-doped graphene covalently grafted with g-C_3N_4, [c] quantum dots of graphitic carbon nitride (g-C_3N_4) and Fe_3O_4 nanoparticles were decorated on MoS_2 nan.

References

1. Long, B.; Yan, G.; He, H.; Meng, S. Porous and Few-Layer Carbon Nitride Nanosheets via Surface Steam Etching for Enhanced Photodegradation Activity. *ACS Appl. Nano Mater.* **2022**, *5*, 7798–7810, doi:10.1021/acsanm.2c00820.
2. Xu, R.; Li, J.; Sui, G.; Zhuang, Y.; Guo, D.; Luo, Z.; Liang, S.; Yao, H.; Wang, C.; Chen, S. Constructing Supramolecular Self-Assembled Porous g-C3N4 Nanosheets Containing Thiophene-Groups for Excellent Photocatalytic Performance under Visible Light. *Applied Surface Science* **2022**, *578*, 152064, doi:10.1016/j.apsusc.2021.152064.
3. Zhang, Q.; Yang, F.; Zhou, S.; Bao, N.; Xu, Z.; Chaker, M.; Ma, D. Broadband Photocatalysts Enabled by 0D/2D Heterojunctions of near-Infrared Quantum Dots/Graphitic Carbon Nitride Nanosheets. *Applied Catalysis B: Environmental* **2020**, *270*, 118879, doi:10.1016/j.apcatb.2020.118879.
4. Zhang, X.; Jia, X.; Duan, P.; Xia, R.; Zhang, N.; Cheng, B.; Wang, Z.; Zhang, Y. V2O5/P-g-C3N4 Z-Scheme Enhanced Heterogeneous Photocatalytic Removal of Methyl Orange from Water under Visible Light Irradiation. *Colloids and Surfaces A: Physicochemical and Engineering Aspects* **2021**, *608*, 125580, doi:10.1016/j.colsurfa.2020.125580.
5. Kampalapura Swamy, C.; Hezam, A.; Mavinakere Ramesh, A.; Habbanakuppe Ramakrishnegowda, D.; K. Purushothama, D.; Krishnegowda, J.; Kanchugarakoppal S., R.; Shivanna, S. Microwave Hydrothermal Synthesis of Copper Induced ZnO/gC3N4 Heterostructure with Efficient Photocatalytic Degradation through S-Scheme Mechanism. *Journal of Photochemistry and Photobiology A: Chemistry* **2021**, *418*, 113394, doi:10.1016/j.jphotochem.2021.113394.
6. Liu, R.; Yang, W.; He, G.; Zheng, W.; Li, M.; Tao, W.; Tian, M. Ag-Modified g-C3N4 Prepared by a One-Step Calcination Method for Enhanced Catalytic Efficiency and Stability. *ACS Omega* **2020**, *5*, 19615–19624, doi:10.1021/acsomega.0c02161.
7. Dou, Q.; Hou, J.; Hussain, A.; Zhang, G.; Zhang, Y.; Luo, M.; Wang, X.; Cao, C. One-Pot Synthesis of Sodium-Doped Willow-Shaped Graphitic Carbon Nitride for Improved Photocatalytic Activity under Visible-Light Irradiation. *Journal of Colloid and Interface Science* **2022**, *624*, 79–87, doi:10.1016/j.jcis.2022.05.085.
8. Liu, F.; Nguyen, T.-P.; Wang, Q.; Massuyeau, F.; Dan, Y.; Jiang, L. Construction of Z-Scheme g-C3N4/Ag/P3HT Heterojunction for Enhanced Visible-Light Photocatalytic Degradation of Tetracycline (TC) and Methyl Orange (MO). *Applied Surface Science* **2019**, *496*, 143653, doi:10.1016/j.apsusc.2019.143653.
9. Huang, Z.; Jia, S.; Wei, J.; Shao, Z. A Visible Light Active, Carbon–Nitrogen–Sulfur Co-Doped TiO 2 /g-C 3 N 4 Z-Scheme Heterojunction as an Effective Photocatalyst to Remove Dye Pollutants. *RSC Advances* **2021**, *11*, 16747–16754, doi:10.1039/D1RA01890F.
10. Santha kumar, K.; Vellaichamy, B.; Paulmony, T. Visible Light Active Metal-Free Photocatalysis: N-Doped Graphene Covalently Grafted with g-C3N4 for Highly Robust Degradation of Methyl Orange. *Solid State Sciences* **2019**, *94*, 99–105, doi:10.1016/j.solidstatesciences.2019.06.003.
11. G-C3N4 Quantum Dot Decorated MoS2/Fe3O4 as a Novel Recoverable Catalyst for Photodegradation of Organic Pollutant under Visible Light | Journal of Materials Science: Materials in Electronics Available online: https://link.springer.com/article/10.1007/s10854-021-06790-w (accessed on 29 November 2023).
